# High Expression of IGFBP7 in Fibroblasts Induced by Colorectal Cancer Cells Is Co-Regulated by TGF-β and Wnt Signaling in a Smad2/3-Dvl2/3-Dependent Manner

**DOI:** 10.1371/journal.pone.0085340

**Published:** 2014-01-10

**Authors:** Cui Rao, Shan-Li Lin, Wen-Jing Ruan, Huan Wen, Dan-Ju Wu, Hong Deng

**Affiliations:** 1 Department of Pathology, School of Medicine, Zhejiang University, Hangzhou, P. R. China; 2 Department of Pathology, Sir Run Run Shaw Hospital, School of Medicine, Zhejiang University, Hangzhou, P. R. China; University of Birmingham, United Kingdom

## Abstract

Fibroblasts in the tumor microenvironment are a key determinant in cancer progression and may be a promising target for cancer therapy. Insulin-like growth factor binding protein 7 (IGFBP7) is known as a tumor suppressor in colorectal cancer (CRC). The present study investigated the inductive mechanism of IGFBP7 expression in fibroblasts by supernatant from the CRC cell line, SW620. The results showed that the expression of IGFBP7 was up-regulated in the fibroblasts when treated with SW620 supernatant and exogenous TGF-β1. The IGFBP7 induced by SW620 supernatant or TGF-β1 was partially inhibited by the TGF-β1 specific antibody AF and TGF-β1 receptor antagonist SB431542. The Wnt signaling-targeted genes, c-Myc, CCND1 and the proteins Dvl2/3, were all up-regulated in fibroblasts expressing high levels of IGFBP7, and the up-regulation could be inhibited both by the Wnt signaling antagonist Dickkopf-1 (DKK1) and by the TGF-β1 receptor antagonist SB431542. In conclusion, CRC cells promote the high expression of IGFBP7 in fibroblasts, most likely through the co-regulation of TGF-β and Wnt signaling in a Smad2/3-Dvl2/3 dependent manner. Taken together, these data suggest that the fibroblasts could be a novel therapeutic target in tumor therapy.

## Introduction

Colorectal cancer (CRC) is one of the most malignant tumors threatening human survival world-wide, ranking third among all malignant tumors [1]. The invasion and metastasis of tumor cells are the main cause of death. Most research has focussed on the tumor itself, yet the important role of the microenvironment in tumor progression has also come to be recognized.

A tumor can be thought of as a wound that does not heal, its invasion and metastasis are not only determined by the cancer cells, but also by the tumor stroma [2]. Micro-metastases form long before a tumor can be diagnosed, and tumor-stroma interaction plays an important role during tumor progression. Fibroblasts are one of the most important cell components of the tumor stroma, where they always acquire an activated phenotype [3]. Just as in fibrosis, fibroblasts in tumors remain persistently activated; they secrete and modulate the extracellular matrix (ECM) in the stroma which plays an important role during tumor progression [3]–[5].

IGFBP7 is a secreted protein which is known to be a tumor suppressor in breast, brain, colon, lung, liver and pancreatic cancers [6]–[11]. It is a cell-adhesive glycoprotein of ∼30 kD [12]. In vivo, different expression patterns of IGFBP7 are found in different tumor types. IGFBP7 expression is low in glioblastoma, lung cancer, pancreatic cancer and liver cancer [6], [9], [10], [13], while both increased and decreased expression of IGFBP7 have been reported in breast and prostate cancer [14]–[17]. These findings suggest that the role of IGFBP7 in tumor cells is complex, though studies on IGFBP7 in tumor stroma cells themselves are rare. In one report, IGFBP7 was found to promote angiogenesis in endothelial cells [18], though the exact role of IGFBP7 in fibroblasts is still unknown.

During tumor-stroma interactions, fibroblasts may be affected by paracrine signaling generated by cancer cells. Among these signalings, TGF-β is thought to be the most potent one. TGF-β-activated fibroblasts create an important pro-invasion and pro-angiogenesis niche for tumor development [19]–[21]. It has been reported that TGF-β signaling acts mainly through the Smad pathways [22]. Upon activation, TGF-β ligand binds to the TGF-β receptor I/II (TβRI/II), and the phosphorylated TβRI recruits and phosphorylates receptor-regulated Smads (R-Smads). The TβRII-ALK5 complex activates Smad2/3, while the TβRII–ALK1 complex activates Smad1/5/8. Once activated, R-Smads phosphorylate and form complexes with Smad4, and move into the nucleus to regulate transcriptional activity [23].

In addition to TGF-β signaling, Wnt signaling also plays an important role in CRC development. Reports have pointed out that ∼90% of CRC cases are due to mutations of the Wnt signaling pathway [24]. Upon canonical Wnt activation, Wnt ligands bind to Frizzled receptors and LRP (low-density lipoprotein receptor-related protein) to promote the phosphorylation of LRP in a Dvl-dependent manner [25], [26]. Then p-LRP recruits Axins from the degradation complex to the cell membrane, helping β-catenin to escape degradation. The accumulated β-catenin moves from the cytoplasm to the nucleus, and forms a transcription activation complex with TCF/LEF. The TCF/LEF-β-catenin nuclear complex activates the transcription of Wnt signaling target genes such as c-Myc, CCND1, FGF20, DKK1 and WISP1, which regulate cell proliferation and differentiation [26]–[29].

In this study, we used the supernatant from SW620, a CRC cell line which is derived from the metastatic tumor of a colorectal carcinoma, and HELF which is one of the canonical fibroblast cell lines. The fact that both SW620 and HELF are IGFBP7 negative cell lines makes interpretation of our findings more unambiguous. The purpose of this study is to examine the expression pattern of IGFBP7 in fibroblasts and the mechanism behind it.

## Materials and Methods

### Reagent and antibodies

TGF-β1 recombinant protein was purchased from PeproTech, USA. AF (TGF-β1 neutralizing antibody) and DKK1 recombinant protein were purchased from R&D, USA. SB431542 (TGF-β/ALK5/Smad2 inhibitor) was obtained from Sigma, USA. The commercial sources of primary antibodies were as follows: rabbit anti-phospho-Smad2 (Ser465/467), mouse anti-Smad2, rabbit anti-β-catenin, rabbit anti-TβRII and rabbit anti-Wnt Signaling Antibody Sampler Kit, Cell Signaling Technology, USA; Rabbit anti-β-actin antibody, Santa Cruz, USA. Secondary antibodies for Odyssey were purchased from Li-COR, USA.

### Cell cultures

Fibroblast (HELF) and CRC cell line SW620 were both cultured in RPMI1640 (Gibco, USA) supplemented with Penicillin-Streptomycin Solution (100 U/ml penicillin, 0.1 mg/ml streptomycin, Gibco, USA), 10% heat-inactivated fetal bovine serum, 50% supernatant of CRC cell line SW620. Culture medium was changed daily for 3 days, for both cell lines. All the cell lines were maintained at 37°C in a 5% CO_2_ atmosphere.

### Preparation of SW620 supernatant

CRC cell line SW620 was plated in culture flasks (8×10^5^ cells) in RPMI1640 supplemented with Penicillin-Streptomycin Solution (as above), and 10% heat-inactivated fetal bovine serum (Gibco, USA). All the cell lines were maintained at 37°C in a 5% CO_2_ atmosphere. The supernatant (SW620-S) was collected after 2 days, filtered with a 0.22 µm filter membrane (Millipore, Ireland) and stored at –80°C.

### Cell treatment

Fibroblasts grown in culture flasks were washed with PBS twice then replaced by RPMI1640 alone or 50% RPMI1640 supplemented with 50% fresh SW620-S, different concentrations of recombinant TGF-β1 protein, with or without AF, SB431542 for various periods of time; the treatments were refreshed every 3 days, and the expression of IGFBP7 was detected by Quantitative real-time PCR, RT-PCR and Western blot analysis.

### Quantitative real-time PCR (Q-PCR) and RT-PCR

Total RNA from treated or untreated fibroblasts was extracted with TRIzol Reagent (Invitrogen, USA) and reverse transcribed into cDNA using Superscript II reverse transcriptase (TaKaRa, Japan). Q-PCR reactions were performed with SYBR Premix Ex Taq (TaKaRa, Japan) on the ABI 7900HT Fast Real-Time PCR System (Applied Biosystems, USA). Primers used for Q-PCR were designed according to the GeneBank (Table S1). The Q-PCR conditions were as follows: 10 seconds denaturation at 95°C, 5 seconds denaturation at 95°C, 30 seconds annealing extension at 60°C for 40 cycles. Fluorescence was detected at the end of each phase. The transcript of IGFBP7 was normalized to the transcript level of housekeeping gene GAPDH. RT-PCR reactions were performed with Taq (TaKaRa, Japan). The RT-PCR conditions were as follows: 5 minutes denaturation at 94°C, 30 seconds denaturation at 94°C, 30 seconds annealing at 59°C, 30 seconds extension at 72°C for 30 cycles, 5 minutes extension at 72°C. The transcript of IGFBP7 was normalized to the transcript level of housekeeping gene GAPDH.

### Western blot analysis

Fibroblasts were lysed in RIPA buffer. The cell lysates were centrifuged at 1.33×10^5^ rpm for 1 hour at 4°C, and protein content in the supernatant was measured using BCA protein assay. 50 µg of total protein extract was loaded into a 10% SDS-PAGE gel and then proteins were transferred to nitrocellulose membrane (BIO-RAD, USA). The membrane was blocked with 5% skim milk TBST (25 mM Tris-HCl, pH 7.5, 150 mM NaCl, 0.05% v/v Tween-20) for 2 hours at room temperature and incubated with primary antibody (1∶1000) overnight at 4°C. After washing with TBST 3 times, blots were probed with secondary antibody (1∶5000) for 1 hour at room temperature. β-actin was used to normalize the amount of the protein loaded sample. Immunoblots were scanned with Odyssey (LI-COR, USA). Semiquantitative evaluation of the bands was performed by densitometric analysis with ImageJ software.

### Immunofluorescence microscopy

Cells on coverslips were fixed in cold acetone for 10 minutes. Coverslips with cells then were washed with PBS 3 times for 5 minutes at room temperature, then permeabilized in 0.05% triton X-100 for 20 minutes and blocked with 10% normal bovine serum for 30 minutes. Cells were incubated with primary antibodies diluted in PBS overnight at 4°C. Coverslips were washed in PBS before incubation for 1 hour with secondary antibodies and 20 minutes with DAPI (1:5000; Invitrogen). Images were taken by a Zeiss LSM510 confocal microscope.

### Data analysis

Results were expressed as mean ± S.E.M. of 3 experiments. The unpaired t-test was used for single comparisons of groups with equal variance and normal distribution. Analysis of variance (ANOVA) followed by Newman-Keuls posttest was used to compare multiple data to each group. Statistical analysis was performed using the Prism software (GraphPad, USA). *P* values less than 0.05 were considered statistically significant.

## Results

### High IGFBP7 expression in fibroblasts is induced by SW620-S

**Figure 1:**
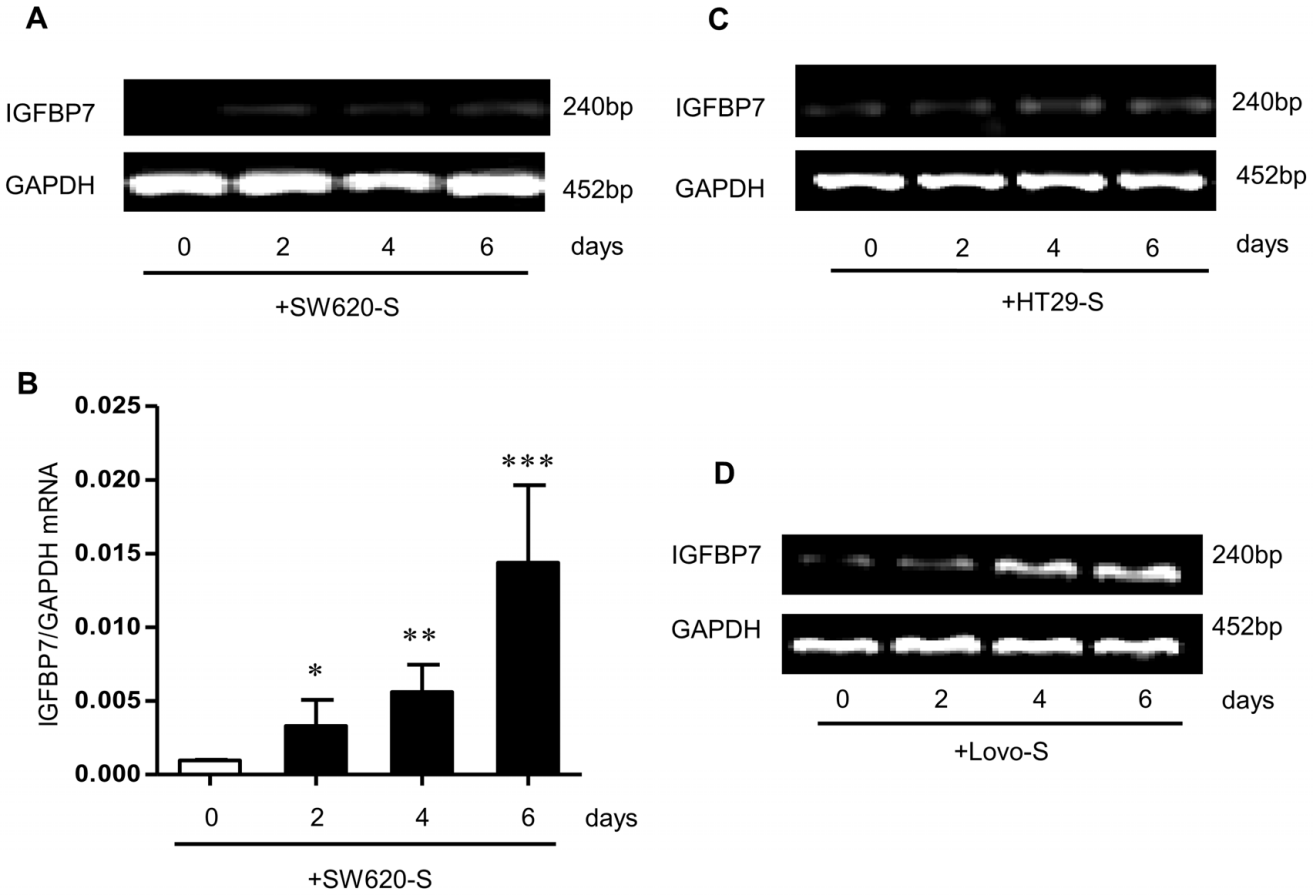
SW620-S induces high IGFBP7 expression in fibroblasts. A. B. IGFBP7 mRNA expression determined by RT-PCR (A) and Q-PCR (B) in fibroblasts exposed to SW620-S for 0, 2, 4 and 6 days respectively. C. D. IGFBP7 mRNA expression determined by RT-PCR in fibroblasts exposed to HT29-S (C) and Lovo-S (D) for 0, 2, 4 and 6 days. The mRNA level was normalized to that of GAPDH in the same cell extracts. *P <0.05 between SW620-S-treated fibroblasts and control group (0 day).

Given the tumor-suppressor role of IGFBP7 in CRC cell lines [30] and the high expression in the invasive front of CRC tumors (unpublished results), the present findings showed that SW620-S induced IGFBP7 expression in fibroblasts, following the incubation of fibroblasts in the presence of SW620-S for various lengths of time. The results revealed that SW620-S substantially up-regulated the level of IGFBP7, compared with the control group. Fibroblasts exposed to SW620-S showed a time-dependent up-regulation of IGFBP7 mRNA (Figure 1A and 1B). In addition, the high IGFBP7 expression was also detected in fibroblasts treated with the supernatant from the other two CRC cell lines HT29 and Lovo (Figure 1C and 1D, Figure S1).

### TGF-β1 in SW620-S induces IGFBP7 expression in fibroblasts

**Figure 2:**
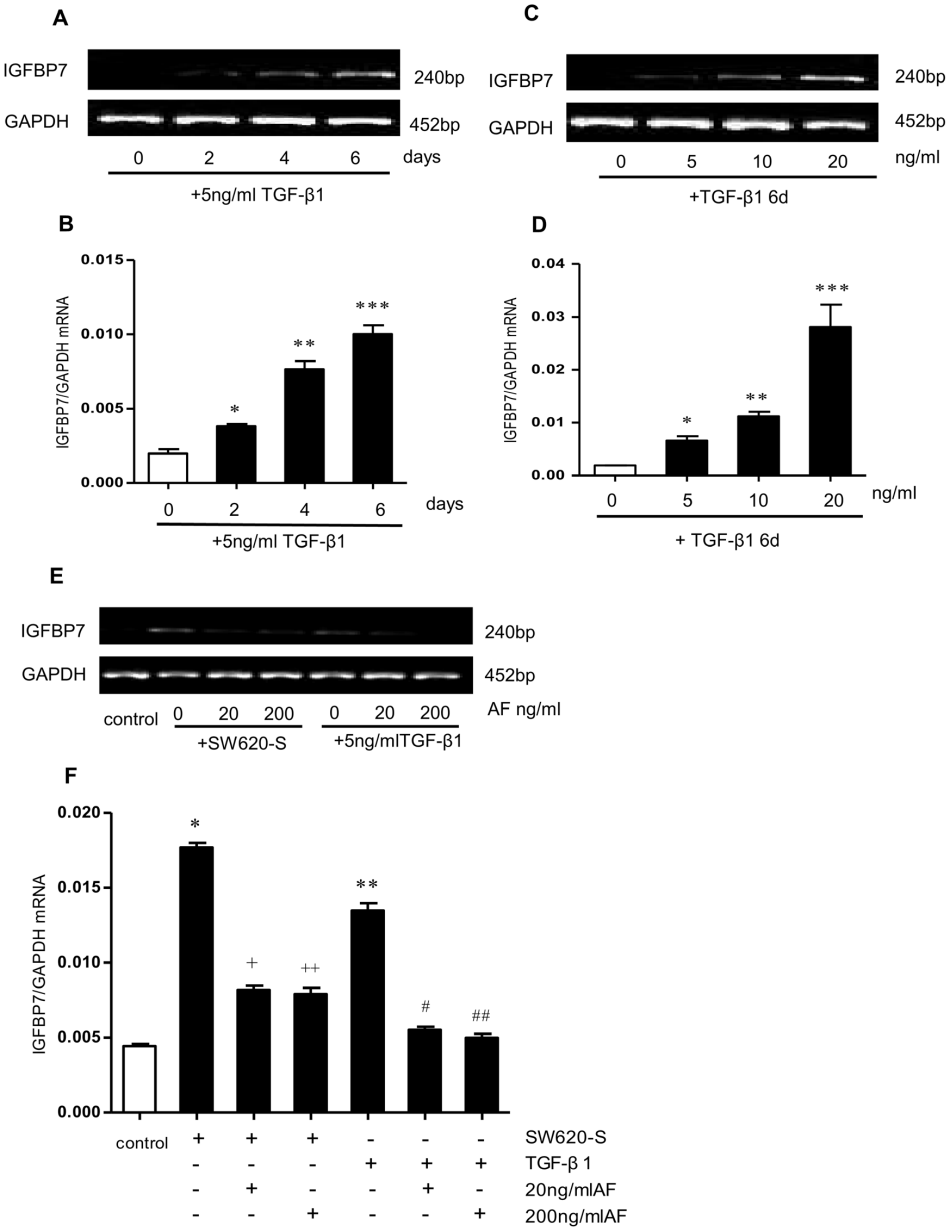
TGF-β1 in SW620-S is the main factor inducing high IGFBP7 expression in fibroblasts. A. B. IGFBP7 mRNA expression determined by RT-PCR (A) and Q-PCR (B) in fibroblasts exposed to RPMI1640 or 5 ng/ml TGF-β1 for 0, 2, 4 and 6 days. C. D. IGFBP7 mRNA expression in fibroblasts exposed to 0, 5, 10 and 20 ng/ml TGF-β1 for 6 days determined by RT-PCR (C) and Q-PCR (D). E. F. Fibroblasts were treated with SW620-S or 5 ng/ml TGF-β1 in the presence of 0, 20, 200 ng/ml AF for 6 days, IGFBP7 mRNA in fibroblasts was detected by RT-PCR (E) and Q-PCR (F). The mRNA level was normalized to that of GAPDH in the same cell extracts. *P <0.05 between SW620-S/TGF-β1-treated fibroblasts and control group. ^+^ *P* <0.05 between SW620-S-treated fibroblasts with or without 20 ng/ml AF. ^++^ *P*<0.05 between SW620-S-treated fibroblasts with or without 200 ng/ml AF. ^#^ *P* <0.05 between TGF-β1-treated fibroblasts with or without 20 ng/ml AF. ^##^ *P*<0.05 between TGF-β1-treated fibroblasts with or without 200 ng/ml AF.

Previous study of the factors secreted by CRC cell lines have shown that cancer cells secrete TGF-β1 during tumor-stroma interactions [31]. On the supposition that the IGFBP7 induction was due to TGF-β1, fibroblasts were exposed to 5 ng/ml of exogenous TGF-β1; they showed a time-dependent increase in IGFBP7 mRNA (Figure 2A and, 2B). Further, fibroblasts exposed to different concentrations of exogenous TGF-β1 ranging from 5 ng/ml to 20 ng/ml for 6 days showed a dose-dependent increase in IGFBP7 mRNA (Figure 2C and 2D). The data showed that the expression of IGFBP7 is not only dependent on time but also on the concentration of TGF-β.

To verify that TGF-β1 was the protein secreted by CRC cells responsible for IGFBP7 induction, we used immunodepletion with a TGF-β1-specific antibody (20 ng/ml AF) to remove TGF-β1 from the SW620-S. This immunodepleted SW620-S failed to induce IGFBP7 in fibroblasts (Figure 2E). Fibroblasts treated with SW620-S or TGF-β1 in the presence of AF partially inhibited the IGFBP7 induced by SW620-S or TGF-β1 alone, which indicated that TGF-β1 was the main, but not the only, factor that induced IGFBP7 (Figure 2F).

### High IGFBP7 expression in fibroblasts is mainly through TGF-β/ALK5/Smad2 signaling pathway

**Figure 3:**
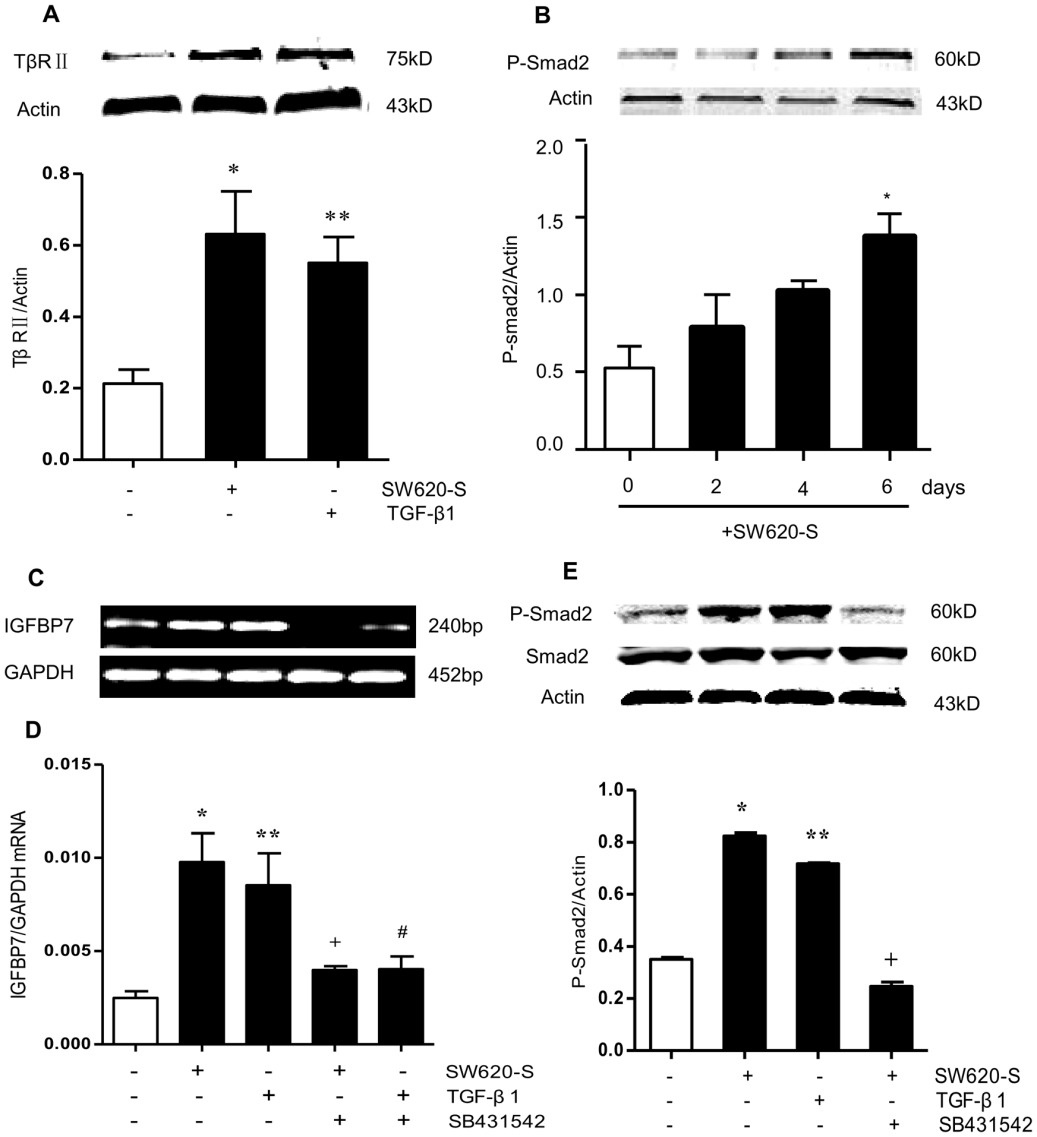
High expression of IGFBP7 in fibroblasts is mainly through the TGF-β/ALK5/Smad2 pathway. A. The expression of TβRII was detected by Western blot. B. Fibroblasts were exposed to SW620-S for 0, 2, 4 and 6 days, and p-Smad2 was detected by Western blot. The protein levels were normalized to that of β-actin in the same cell extracts. C.D. Fibroblasts were exposed to SW620-S or TGF-β1 in the presence of 10 µM SB431542 for 6 days, and IGFBP7 mRNA expression was detected by RT-PCR (C) and Q-PCR (D). The mRNA level was normalized to that of GAPDH in the same cell extracts. E. Fibroblasts were exposed to SW620-S or TGF-β1 with 10 µM SB431542 for 6 days, and the expression of Smad2, p-Smad2 (E) was detected by Western blot. The protein levels were normalized to that of β-actin in the same cell extracts. ^*^ *P*<0.05 between SW620-S/TGF-β1-treated fibroblasts and control group. ^+^ *P*<0.05 between SW620-S-treated fibroblasts with or without SB431542, ^#^ *P*<0.05 between TGF-β1-treated fibroblasts with or without SB431542.

In TGF-β signaling, the TβRII-ALK5 complex phosphorylates Smad2/3. In order to determine whether the induction of IGFBP7 was through the Smad2-dependent signaling, SB431542 (a selective antagonist of TGF-β/ALK5/ Smad2 signaling) was added to fibroblasts. In fibroblasts exposed to SW620-S or exogenous TGF-β1 with 10 µM SB431542, the IGFBP7 induction was partially inhibited (Figure 3C and 3D). High expression of P-Smad2 was detected in fibroblasts treated with SW620-S for various lengths of time. This up-regulation of P-Smad2 was time-dependent, reaching a peak on day 6 (Figure 3B). The levels of p-Smad2 (Figure 3E) and TβRII (Figure 3A) increased by SW620-S or exogenous TGF-β1 were decreased to the normal level by SB431542, which indicated that this up-regulation was through the TβRII-ALK5-Smad2/3 pathway.

### IGFBP7 up-regulation in fibroblasts is partially through the activation of canonical Wnt signaling pathway

**Figure 4:**
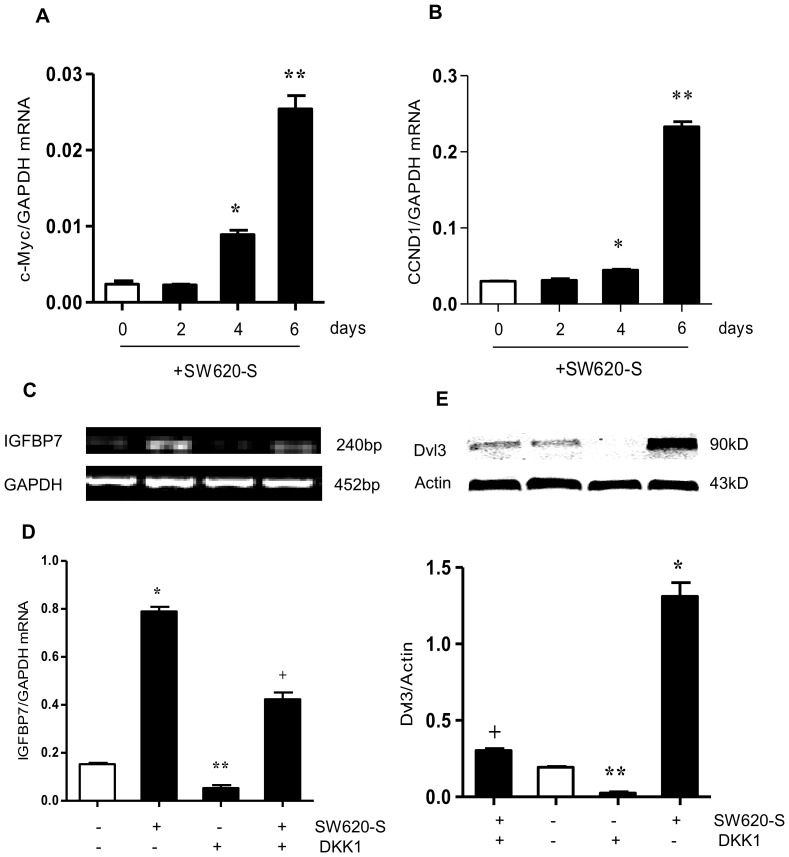
IGFBP7 up-regulation in fibroblasts is partially through the activation of Wnt signaling. A. B. Fibroblasts were treated with SW620-S for 0, 2, 4 and 6 days, and the mRNA expression of the Wnt signaling target genes c-Myc (A) and CCND1 (B) mRNA expression was assessed by Q-PCR. C-E. Fibroblasts were treated with SW620-S in the presence of 50 ng/ml DKK1 (Wnt antagonist) for 6 days, and IGFBP7 mRNA expression was detected by RT-PCR(C) and Q-PCR (D). The mRNA expression was normalized to that of GAPDH in the same cell extracts. The Wnt signaling protein Dvl3 was detected in fibroblasts by Western blot (E). Protein expression was normalized to that of β-actin in the same cell extract. *P<0.05 between SW620-S-treated fibroblasts and control group. **P<0.05 between DKK1-treated fibroblasts and control group. ^+^ *P*<0.05 between SW620-S-treated fibroblasts with or without DKK1.

Since Wnt signaling pathway regulates the activation of fibroblasts, we set out to determine whether Wnt, especially the canonical Wnt signaling pathway, was another regulator. Fibroblasts treated with SW620-S showed high expression of c-Myc and CCND1, the downstream target genes of Wnt signaling, in a time-dependent manner. The canonical Wnt signaling protein β-catenin detected by Western blot (Figure S2A) was increased and activated as shown by immunofluorence microscopy (Figure S2B) which indicated that canonical Wnt signaling was activated during IGFBP7 up-regulation (Figure 4A and 4B).

Having shown that Wnt signaling was activated, we then investigated the relationship between this activation and the high expression of IGFBP7. DKKs are secreted glycoproteins that are known to antagonize canonical Wnt signaling [32]. When fibroblasts were treated with DKK1, the high expression of IGFBP7 induced by SW620-S was partially inhibited (Figure 4C and 4D). The Wnt signaling proteins Dvl2/3 detected by Western blot were inhibited by DKK1 (Figure 4E), suggesting that the up-regulation of IGFBP7 was closely associated with the canonical Wnt signaling pathway. Therefore, the factors responsible for the high expression of IGFBP7 may be TGF-β1 and Wnt ligands like Wnt3a that activated the TGF-β and the Wnt signaling pathway.

### TGF-β and Wnt canonical signaling pathway co-operate in regulating the high expression of IGFBP7 in fibroblasts

**Figure 5:**
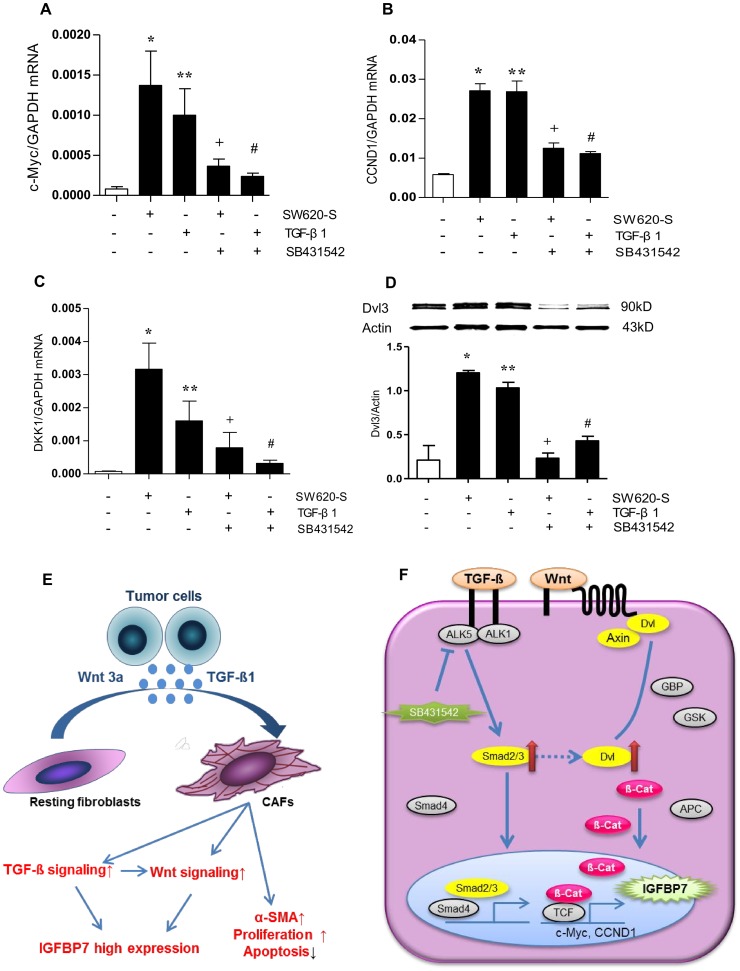
TGF-β and canonical Wnt signaling co-operate in regulating the high expression of IGFBP7 in fibroblasts. A-D. Fibroblasts were exposed to SW620-S or TGF-β1 in the presence of 10 µM SB431542 for 6days, and the mRNA expression of the Wnt signal target genes c-Myc (A), CCND1 (B) and DKK1 (C) were assessed by Q-PCR. mRNA expression was normalized to that of GAPDH in the same cell extracts. The expression of Dvl3 was detected by Western blot (D). Protein expression was normalized to that of β-actin in the same cell extract. *P<0.05 between SW620-S-treated fibroblasts and control group. **P<0.05 between TGF-β1-treated fibroblasts and control group.^ +^ *P*<0.05 between SW620-S-treated fibroblasts with or without SB431542. ^#^ *P*<0.05 between TGF-β1-treated fibroblasts with or without SB431542. E. Model of high expression of IGFBP7 induced by the tumor cell-fibroblast interactions. F. TGF-β signal activation up-regulates Smad2/3 and Dvl2/3, accompanied by up-regulation of the Wnt target genes c-Myc, CCND1 and DKK1, such that IGFBP7 is over-expressed.

In order to clarify the relationship between TGF-β and Wnt signaling pathway, we treated fibroblasts with TGF-β1, and found that it also increased the level of c-Myc, CCND1 and DKK1; moreover, this up-regulation was partially inhibited by SB431542 (Figure 5A-5C). These results were seen at the protein level, as Dvls proteins are positive mediators of Wnt signaling located downstream of the Frizzled receptors and upstream of β-catenin. Dvl3 was up-regulated by TGF-β1 treatment (Figure 5D), indicating that Wnt signaling was activated during the interaction and this change was reversed by the TGF-β antagonist SB431542, suggesting that with TGF-β signaling activation, Wnt signaling was also activated. We therefore found the existence of a novel cross-talk between Wnt and TGF-β signaling which was Dvl2/3-Smad2/3-dependent.

## Discussion

These experiments have demonstrated that the supernatant from the SW620 cell line (SW620-S) induced IGFBP7 in fibroblasts mainly through the co-regulation of TGF-β/ALK5/Smad2 signaling and canonical Wnt signaling. This is the first evidence of the mechanism underlying the high IGFBP7 expression in fibroblasts during tumor-stroma interactions. Our hypothesis is that IGFBP7 is one of the downstream target genes of TGF-β/Smad2 and canonical Wnt signaling, though the exact position of IGFBP7 still needs further investigation.

In this study, we have shown that CRC cells induced a high expression of IGFBP7 (Figure 5E). As IGFBP7 is a tumor suppressor, most research on IGFBP7 has focussed on the tumor cells themselves. In a tumor xenograft model, for example, the over-expression of IGFBP7 inhibited the growth of melanoma [33]. Why then do the tumor cells secrete factors which induce IGFBP7 which in turn suppresses their own growth? This may be the result of the resistance of fibroblasts to the tumor cells. What is the significance of the high expression of IGFBP7 in fibroblasts? At present, this is difficult to assess, because there is little research on the role of IGFBP7 in tumor stroma. IGFBP7 was found highly expressed in the vessels of glioma [34]; it can interact with extracellular matrix protein to induce the adhesion and migration of endothelial cells [35], [36]. Pen and colleagues also reported that IGFBP7 in endothelial cells can induce angiogenesis [18]. Others found that IGFBP7 probably participates in the activation and proliferation of fibroblasts [37]. These observations suggest that IGFBP7 in tumor cells in stroma may play a completely different role from IGFBP7 in the stromal cells of tumors. Our unpublished data showed that IGFBP7 may correlate with the activation of fibroblasts, but the detailed significance of its high expression in fibroblasts is still unknown and requires further investigation.

Our results showed that IGFBP7 expression in fibroblasts is closely related with the TGF-β secreted by CRC cells. Also, the expression of IGFBP7 is not only time-dependent but also dose-dependent with regard to TGF-β. The antibody and inhibitor of TGF-β confirmed this result. It has been reported that TGF-β signaling has also regulated the expression of IGFBP7 in brain endothelial cells [18], a further example of a stroma cell compartment. And we know, TGF-β/Smad3 signaling has been regarded as tumor suppressor during tumor progression since TGF-β-induced CDKN1A (P16) and CDKN2B (P15) expression is correlated with tumor inhibition [38]. However, other research finds that CTGF and PAI-1, the downstream target gene of TGF-β/Smad3 signaling, is correlated with a high risk of metastasis in breast cancer [39]. It suggests TGF-β/Smad3 may inversely promote the invasion and migration in the late stage of tumor formation [40], [41]. Therefore, we speculate that TGF-β secreted by cancer cells promoting IGFBP7 expression in fibroblasts may lessen the tumor suppressor role for fibroblasts in tumor tissue.

The relationship of IGFBP7 and Wnt signaling has not been investigated, and our study is the first to show that Wnt signaling may regulate the expression of IGFBP7 in fibroblasts. It has been reported that Wnt/β-catenin signaling regulates the differentiation of fibroblasts, which suggests that Wnt/β-catenin signaling is an important regulator of the fibroblast phenotype [42]. This suggests that Wnt signaling may affect IGFBP7 through mediating the differentiation status of fibroblasts.

Wnt signaling is known to be activated when fibroblasts are activated by TGF-β [43]. Here we noticed that Wnt signaling is activated during IGFBP7 induction in fibroblasts by TGF-β. It is strange that except for c-Myc and CCND1, the expression of DKK1 was also up-regulated by TGF-β. We know that DKK1 is a canonical antagonist of Wnt signaling. These published findings are therefore inconsistent with our results. Yet, in addition, we should note that DKK1 is also the target gene of Wnt signaling [28]. This may be a negative feedback mechanism between these two signaling pathways. These two signals may co-operate during IGFBP7 induction in fibroblasts. With regard to how they co-operate: we found that they were connected by Smad2/Dvl3. There are several explanations for the interaction between these two signaling pathways. One mode of interaction between Wnt/β-catenin and TGF-β/Smads signals is the interaction between Smads and β-catenin/TCF4 [44]. Another mode may be by Wnt5a inducing the formation of the MARK2/Dvl3/Smad4 complex [45]. Yet another mode may be Wnt3a promoting a cancer-associated fibroblasts-like phenotype in fibroblasts, partially through the TGF-β/Smad2 signaling activation by a β-catenin-dependent mechanism [46], indicating that Wnt and TGF-β signaling are linked by Smad. From our results, in fibroblasts, the activation of Wnt induced by TGF-β was most likely through the up-regulation of Smad and Dvl3. We therefore conclude that Dvl3 may be a novel point of cross-talk between TGF-β and Wnt signaling pathways in fibroblasts. Smad2/3 and Dvl2/3 may form some kind of compound that could link TGF-β and Wnt signaling (Figure 5F). It has been reported that TGF-β and Wnt signaling co-regulate the determination of mesenchymal stem cell fates in mammary cells, including the induction of cancer stem cells. Therefore, combination of these two signaling pathways may be a mechanism behind some conditions such as fibrotic diseases [47]–[49].

We know that the paracrine signaling molecules secreted by CRC cells during tumor stroma interactions include TGF-β, Wnt and some other factors, and that these may change the neighboring fibroblasts. We also know that fibroblasts in tumor stroma are responsible for the synthesis of MMPs and collagen fibers. MMPs help tumor cells invade the basement membrane, while collagen fibers provide tumor cells with a convenient physical substratum over which to migrate [50]. This is why fibroblasts in tumor stroma have been called “Distant invaders and migrators” [51].

All in all, the tumor-stroma interaction is a complex process. During this process, tumor cells may secrete factors to alter the differentiation of normal fibroblasts, and the changed fibroblasts in return secrete factors affecting the progression of tumor invasion and migration. We have presented data indicating the mechanism of how IGFBP7 may be involved in this interaction, and this interaction, emphasizing the involvement of tumor stromal fibroblasts, could form the focus of a novel therapeutic approach to the management of cancer.

## Supporting Information

Figure S1 **HT29-S and Lovo-S induce high expression of IGFBP7 in fibroblasts.** Semi-quantitative analysis of IGFBP7 mRNA expression level determined by RT-PCR in fibroblasts exposed to HT29-S (A) and Lovo-S (B) for 0, 2, 4 and 6 days. The mRNA level was normalized to that of GAPDH in the same cell extracts. *P<0.05 between HT29-S/Lovo-S-treated fibroblasts and control group (0 day).
(TIF)

Figure S2 **β-Catenin is activated during the up-regulation of IGFBP7 in fibroblasts.** A. Fibroblasts were treated with SW620-S or TGF-β for 6 days and the expression of Wnt signaling protein β-catenin was detected in fibroblasts by Western blot. Protein expression was normalized to that of β-actin in the same cell extract. *P <0.05 between TGF-β1-treated fibroblasts and control group. B. Fibroblasts were treated with SW620-S or TGF-β for 6 days and β-catenin (red) and DAPI (blue) were detected in fibroblasts by immunofluorescence microscopy (Original magnification ×1000).
(TIF)

Table S1 **Q-PCR Primers.**
(DOC)
