# Supplementary figures and images for: High Expression of IGFBP7 in Fibroblasts Induced by Colorectal Cancer Cells Is Co-Regulated by TGF-β and Wnt Signaling in a Smad2/3-Dvl2/3-Dependent Manner
Source: PLoS One. 2014 Jan 10;9(1):e85340. doi: 10.1371/journal.pone.0085340 (PMC3888407; doi:10.1371/journal.pone.0085340)

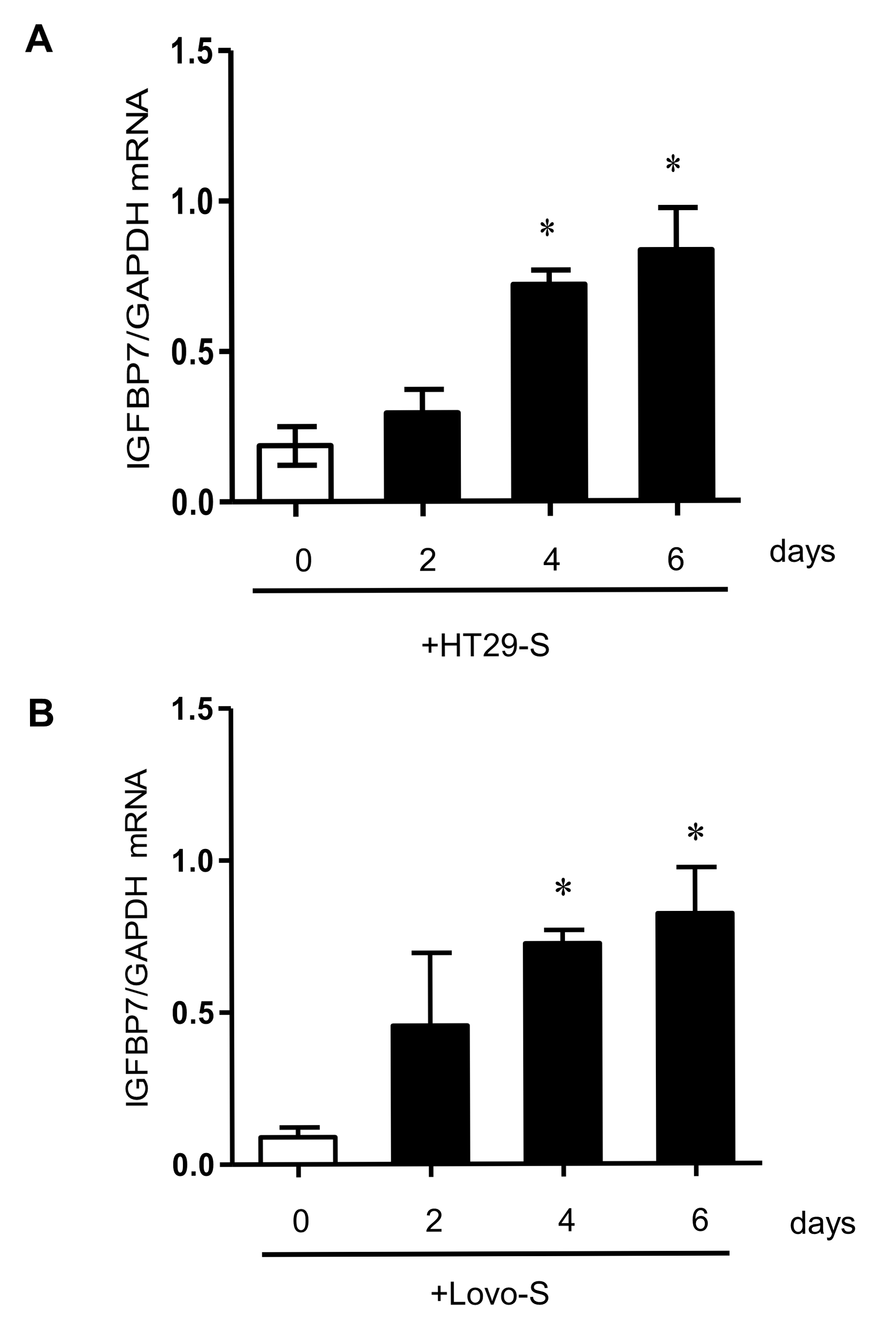

Supplement: Figure S1 — HT29-S and Lovo-S induce high expression of IGFBP7 in fibroblasts. Semi-quantitative analysis of IGFBP7 mRNA expression level determined by RT-PCR in fibroblasts exposed to HT29-S (A) and Lovo-S (B) for 0, 2, 4 and 6 days. The mRNA level was normalized to that of GAPDH in the same cell extracts. *P<0.05 between HT29-S/Lovo-S-treated fibroblasts and control group (0 day). (TIF) [file pone.0085340.s001.tif]

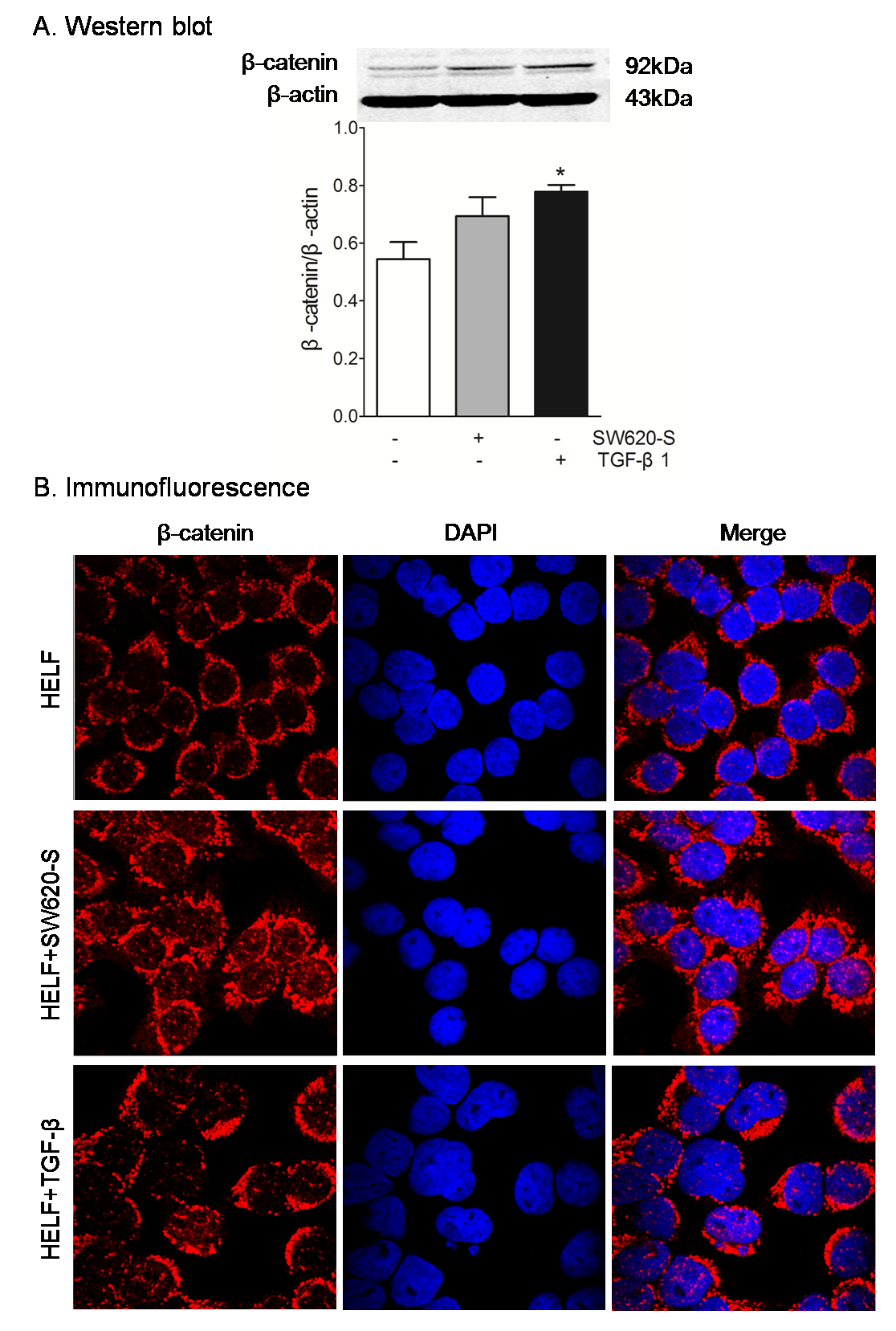

Supplement: Figure S2 — β-Catenin is activated during the up-regulation of IGFBP7 in fibroblasts. A. Fibroblasts were treated with SW620-S or TGF-β for 6 days and the expression of Wnt signaling protein β-catenin was detected in fibroblasts by Western blot. Protein expression was normalized to that of β-actin in the same cell extract. *P <0.05 between TGF-β1-treated fibroblasts and control group. B. Fibroblasts were treated with SW620-S or TGF-β for 6 days and β-catenin (red) and DAPI (blue) were detected in fibroblasts by immunofluorescence microscopy (Original magnification ×1000). (TIF) [file pone.0085340.s002.tif]
