# Supplementary material for: High Expression of IGFBP7 in Fibroblasts Induced by Colorectal Cancer Cells Is Co-Regulated by TGF-β and Wnt Signaling in a Smad2/3-Dvl2/3-Dependent Manner
Source: PLoS One. 2014 Jan 10;9(1):e85340. doi: 10.1371/journal.pone.0085340 (PMC3888407; doi:10.1371/journal.pone.0085340)
Supplement: Table S1 — Q-PCR Primers. (DOC) [file pone.0085340.s003.doc]

**Table S1 Q-PCR Primers**

| **Target gene** | **FWD Primer** | **REV Primer** |
| --- | --- | --- |
| GAPDH | ACCACAGTCCATGCCATCAC | TCCACCACCCTGTTGCTGTA |
| IGFBP7 | CACTGGTGCCCAGGTGTACT | TTGGATGCATGGCACTCATA |
| c-Myc | AGCGACTCTGAGGAGGAACA | TCGCCTCTTGACATTCTCCT |
| CCND1 | AACTACCTGGACCGCTTCCT | CCACTTGAGCTTGTTCACCA |
| DKK1 | TGCCCAGAAAATGAAAAAGG | GTGTATGTGGCAATGCGTTC |
